# Suitability of the woodchuck HCC as a preclinical model for evaluation of intra‐arterial therapies

**DOI:** 10.1002/ame2.12100

**Published:** 2020-02-09

**Authors:** Alexander Y. Kim, Joseph H. Yacoub, David H. Field, Byoung Uk Park, Bhaskar Kallakury, Kyle E. Korolowicz, Stephan Menne

**Affiliations:** ^1^ Department of Radiology Medstar Georgetown University Hospital Washington District of Columbia; ^2^ Department of Pathology and Laboratory Medicine Medstar Georgetown University Hospital Washington District of Columbia; ^3^ Department of Microbiology and Immunology Georgetown University Medical Center Washington District of Columbia

**Keywords:** angiography, animal model, hepatocellular carcinoma, woodchuck

## Abstract

The most commonly used preclinical models of hepatocellular carcinoma (HCC) are limited for use in testing of intra‐arterial therapies such as transarterial chemoembolization and radioembolization. Issues encountered with the more commonly used animal models include dissimilarity in their disease development compared with humans and the size of the vasculature which can make intra‐arterial therapy testing difficult or impossible. Here we describe the suitability of the woodchuck HCC model for testing of intra‐arterial therapies. We describe the techniques for pre‐embolization imaging assessment using CT and MRI, technical tips on performing angiography and embolization, and pathological assessment of treated liver.

## 1 INTRODUCTION

Since two randomized, controlled trials demonstrated an overall survival advantage of transarterial chemoembolization in a select hepatocellular carcinoma (HCC) population,^1^, ^2^ there have been no other randomized trials demonstrating further survival improvement—despite the rapid advances in drug, particle, catheter, and imaging technologies in the intervening 20 years.^3^, ^4^ While reasons behind this discrepancy are complex and likely multifactorial, one contributing factor may be the lack of an appropriate animal HCC model for preclinical testing.

The most commonly used animal models for the study of HCC include mice, rats, and rabbits.^5^, ^6^ Although cost‐effectiveness and availability make these animal models easy to use, their effectiveness in the evaluation of intra‐arterial therapies (IAT) is limited. For example, the size of these animals makes cannulating the vascular system difficult, if not impossible.^7^, ^8^, ^9^ Although vascular access may be possible after surgical cut down, catheters commonly used in humans are often too large to fit into the visceral vessels of these animals. In addition, some of these are xenograft models in whom disease pathogenesis is a poor representation of human HCC.^6^

In contrast, the Eastern woodchuck (*Marmota monax*) develops HCC through a natural, virally induced pathway.^10^, ^11^ After exposure to the woodchuck hepatitis virus (WHV) as neonates, which is a DNA virus akin to the human hepatitis B virus (HBV), an exposed woodchuck can develop HCC over a median period of 24‐32 months with an approximate 6‐month life expectancy thereafter.^10^, ^11^ The similarity of WHV to HBV and the natural development of HCC have made the woodchuck HCC model invaluable in antiviral research.

Given this analogous development of HCC in an animal whose size is sufficient for arterial catheterization, we hypothesized that the woodchuck would be an ideal preclinical animal model for the study of IAT. A prior study has demonstrated the feasibility of performing arterial catheterization in a nontumor woodchuck model.^12^ The purpose of technical note was to describe the imaging parameters of pre‐procedural cross‐sectional imaging, demonstrate the feasibility of performing IAT, and to detail the pathologic assessment after embolization.

## 2 METHODS

### 2.1 Woodchuck HCC model

This animal study was approved by the Institutional Animal Care and Use Committee. Woodchucks received humane care as outlined in the National Institutes of Health Guide for the Care and Use of Laboratory Animals.

Three female woodchucks, ranging in weight between 2.6 and 5.6 kg, were infected with WHV at three days of age. The animals were purchased from a vendor (Northeastern Wildlife, Harrison, ID) after viral infection and maintained in the animal facility at Georgetown University until tumor development. These animals served as the untreated control arm in unrelated experimental studies. Tumor development was monitored by serial ultrasound and liver function enzyme levels, specifically GGT, which has been validated to be an oncogenic biomarker in this animal model.^13^ Free access to food and water was provided until the day of study. Initial sedation was induced by intramuscular injection of ketamine 50 mg/kg mixed with xylazine (1‐5 mg/kg) and maintained for the duration of the study with 1%‐3% isofluorane inhalant via nose cone. Animal sedation was overseen by one of the authors (SM) with 22 years’ experience in woodchuck research. Animals were maintained under sedation for all portions of the study, both imaging and procedural.

### 2.2 Technique for arterial access

Initial arterial access was obtained into the right or left common femoral artery under direct sonographic guidance (LOGIQE9; GE Healthcare, Chicago, IL) with a micropuncture kit (Angiodynamics, Latham, NY) The outer 5Fr transition dilator of the micropuncture kit was used as the vascular access site for delivery of contrast for all pre‐procedural imaging evaluations.

### 2.3 Protocol for CT assessment

CT of the abdomen was performed with a 16‐slice scanner (SOMATOM Emotion 16; Siemens Healthineers, Erlangen, Germany) at 80 kVp with tube current‐time product of 15 mAs. Images were reconstructed with 2‐3 mm section thickness. Pre‐contrast images were acquired prior to the administration of 3 mL intravenous contrast (Omnipaque 350; GE Healthcare, Chicago, IL), delivered by injector at a rate of 0.3 mL/s. Arterial phase, portal venous phase, and delayed images were obtained at approximately 3, 30, and 70 seconds following initiation of the injection.

### 2.4 Protocol for MRI assessment

MRI of the abdomen was performed with a 1.5 Tesla MRI scanner (MAGNETOM Symphony; Siemens Healthineers, Erlangen, Germany). Imaging protocol included axial and coronal single‐shot fast‐spin echo (HASTE), axial single‐shot fast‐spin echo inversion recovery (HASTE IR), axial in/out of phase, diffusion‐weighted imaging, balanced steady‐state free procession (True FISP) and pre‐ and post‐contrast T1‐weighted 3D gradient echo dynamic (VIBE). Intravenous gadolinium (Gadavist, Bayer HealthCare, Whippany, NJ) was administered by a hand injection at a dose of 0.1 mL/kg. Post‐contrast dynamic VIBE sequences were acquired immediately after the injection and repeated every 11.8 seconds for 10 total sequences. A delayed post‐contrast VIBE sequence was acquired at 3 minutes.

### 2.5 Angiography and embolization technique

Angiography was performed through right or left common femoral arterial access under direct sonographic guidance with a linear probe. A 4‐F Soft‐Vu catheter (Angiodynamics, Latham, NY) was advanced over a 0.035″ InQwire (Merit Medical, South Jordan, UT) and positioned in the mid‐abdominal aorta. Angiography was performed with hand injection of approximately 2 mL Isovue 300 (Bracco, Milan, Italy). A 2.8‐F Transcend HI‐FLO microcatheter (Boston Scientific, Marlborough, MA) was advanced coaxially into the common hepatic or hepatic proper artery. Angiography was performed with hand injection of approximately 1‐2 mL of Isovue 300 diluted 50:50 with saline. 3D rotational angiography (Philips Allura, Amsterdam, the Netherlands) was performed with hand injection of a similar volume of half‐dilute contrast. Early arterial images were obtained during arterial injection with a 2‐second delay from the start of the injection and late‐phase images were obtained 10 seconds after the completion of the initial scan. Reconstructed cone‐beam CT images were evaluated on a dedicated workstation.

Embolization was performed using 2 mL of 100‐300 micron LC‐beads (BTG, London, UK) diluted to a total of 20 mL with Isovue 300. The particles were resuspended with 20 and 3 mL syringes connected to a three‐way stopcock and delivered through the microcatheter, which was positioned with its tip in the hepatic proper artery. Infusion was performed until complete stasis was reached.

### 2.6 Technique for pathologic assessment

Following embolization, the animal was euthanized with intracardiac injection of 80‐100 mg/kg phenytoin‐pentobarbital (Beuthanasia‐D solution; Vedco Inc, St. Joseph, MO) and the liver was harvested. The specimens were serially sectioned from anterior to posterior at 1 cm intervals and placed in 4% formaldehyde for immersion fixation. For standardized microscopic evaluation, 15 sections of the liver were taken as follows: nine representative sections of uninvolved (normal) liver parenchyma (anterior, mid, and posterior aspects of left lateral lobe, quadrate lobe, right medial lobe, right lateral lobe, caudate process, and papillary process) and six sections of the tumor (medial, lateral, anterior, posterior, superior, and inferior aspects). The glass slides were prepared according to standardized method (paraffin embedded, sectioned with a microtome and stained with hematoxylin and eosin).

## 3 RESULTS

### 3.1 MRI findings

**Figure 1:**
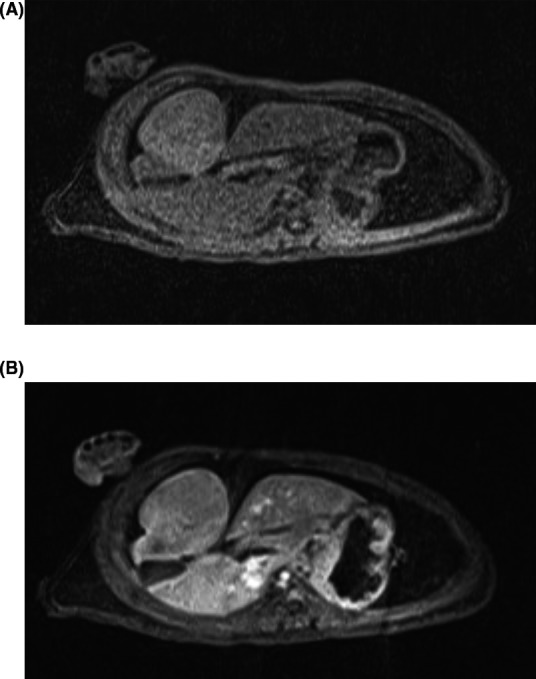
MRI findings. A, T1‐weighted MRI image of a woodchuck HCC demonstrates an exophytic right lobe mass. B, Post‐contrast images demonstrate the lesion to be isoenhancing versus background liver

MRI images from one animal demonstrate HCC lesion with mild intrinsic T1 hyperintensity relative to background liver on pre‐contrast images (Figure 1A). It was isoenhancing relative to background liver on early post‐contrast sequences (Figure 1B) and demonstrated mild washout on the delayed sequences. On T2‐weighted imaging, the tumor was heterogeneous and mildly hypointense compared with background liver parenchyma.

### 3.2 CT demonstrates washout

**Figure 2:**
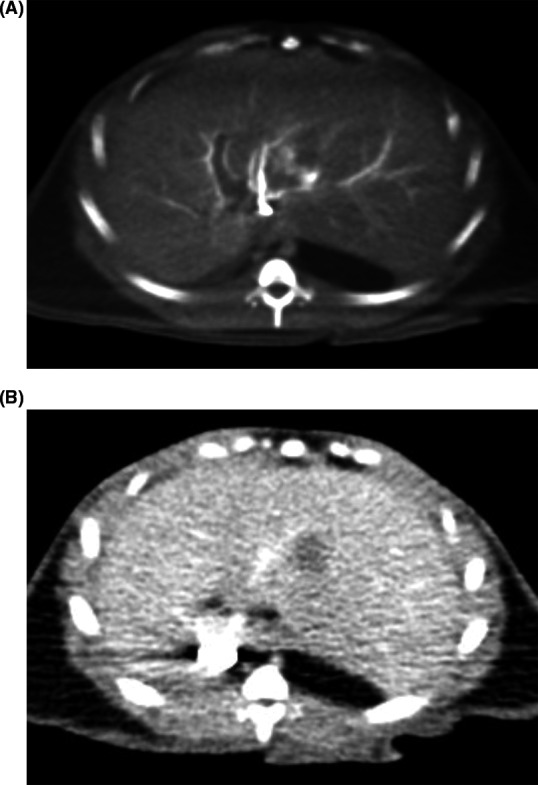
CT findings. A, Post‐contrast CT images demonstrate eccentric enhancement of a left lobe HCC. B, Delayed phase CT images show washout of this mass relative to background liver

CT images from a second animal demonstrate another HCC lesion to be hypodense relative to the remainder of the hepatic parenchyma in the left lobe. On the early post‐contrast images, the portal veins were opacified; contrast in the IVC was refluxed from the right atrium. The hepatic veins were not opacified. The tumor itself was partially hypoenhancing with an eccentric blush of contrast (Figure 2A,B). On the portal venous and delayed phase images, the hypoattenuating portion of the tumor became more conspicuous, with an area of subtle eccentric washout most evident on the delayed phase. While the hypoattenuating portion of the lesion measured 1.1 cm, its overall size was difficult to assess due to indistinct margins.

### 3.3 Identification of tumor feeding artery on angiography

**Figure 3:**
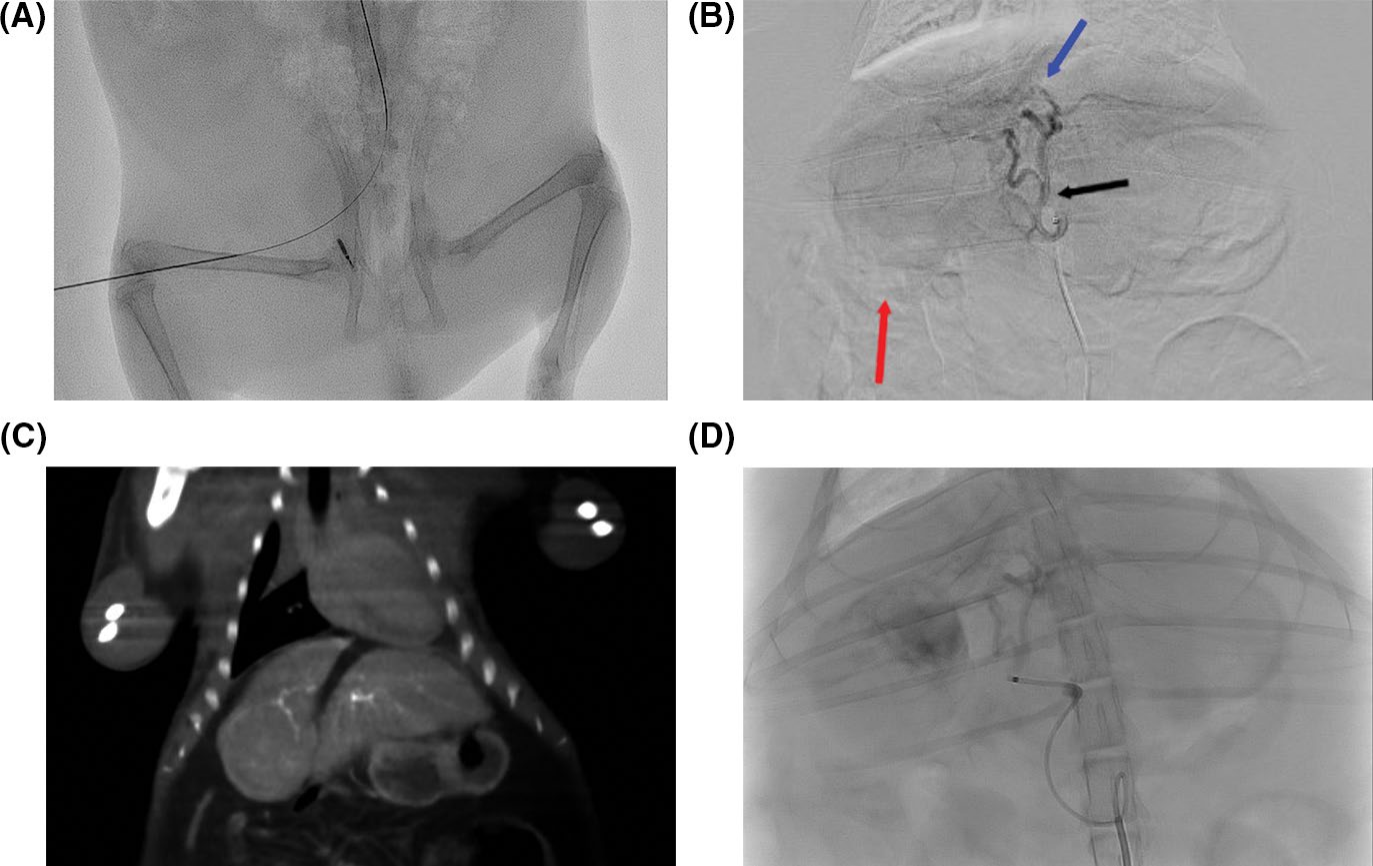
Angiographic findings. A, Spot image after microwire advancement subsequent to obtaining percutaneous arterial access. B, Angiogram with the catheter tip in the common hepatic artery. Black arrow denotes the bifurcation of the right and left hepatic arteries. Red arrow points to outline of the right lobe HCC with peripheral regions of enhancement. Blue arrow points to a hypertrophic and tortuous segment 4 artery which provided the dominant arterial supply to tumor. C, Coronal‐reconstructed CBCT image demonstrating tumor enhancement. The tortuous, hypertrophic arterial feeder is demonstrated in the superior aspect of the tumor. D, Post‐embolization of the tumor from the hepatic proper artery demonstrates contrast retention in the superior aspect of the tumor

Arterial access of the common femoral artery can be obtained under direct ultrasound guidance (Figure 3A). Angiographic images of animal 3 demonstrate a large right lobe lesion with hypertrophic arteries supplying the tumor (Figure 3B). Dual‐phase cone‐beam CT with contrast injection from the hepatic proper artery allows for easy identification of the exophytic tumor with delineation of the vascular tumor supply (Figure 3C). Post‐embolization images demonstrate a heterogeneous pattern of contrast retention in the tumor (Figure 3D).

### 3.4 Pathologic assessment of particle distribution

**Figure 4:**
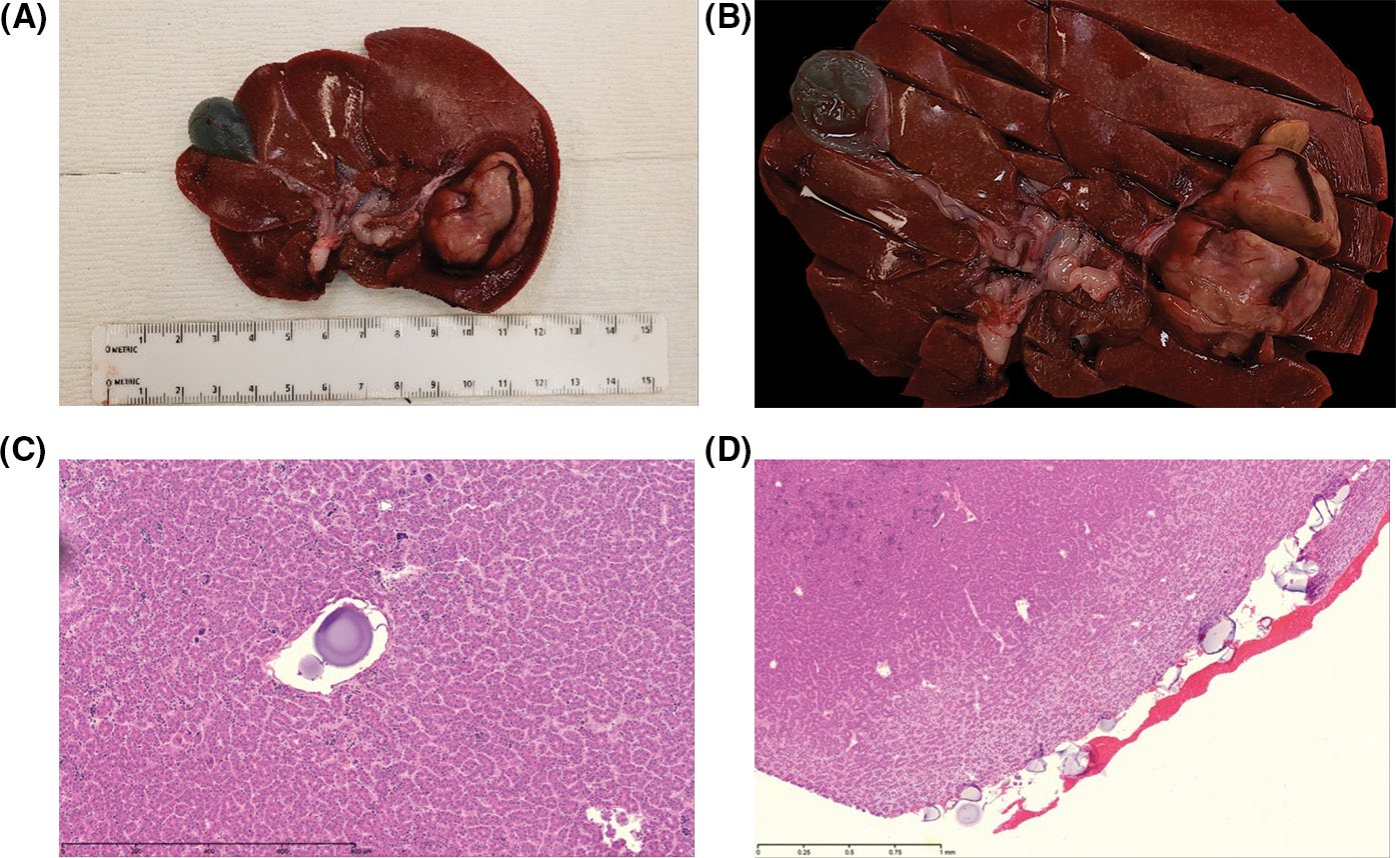
Pathologic findings. A, Gross liver specimen. The tan‐colored exophytic lesion in the left lobe is readily visible. B, Gross liver specimen sectioned in 1 cm intervals, anterior to posterior. C and D, Representative sections of liver demonstrating the presence of embolic particles within tumor

Tumor was grossly identifiable and appears tan compared with the background normal liver (Figure 4A,B). Microscopic evaluation demonstrates heterogeneous distribution of embolic particles (Figure 4C,D).

## 4 DISCUSSION

Identification of an appropriate animal model for testing novel advances in IAT is crucial to our ability to translate these techniques into improvements in patient outcomes. An ideal preclinical model should provide disease development on a scale that readily allows for testing of human‐size products. The woodchuck is an attractive HCC model for exactly these reasons.^11^

The woodchuck develops HCC through a natural, virally induced method, similar to that of humans. The use of imaging protocols as outlined in our study allows for easily replicable CT and MRI tumor assessment.

As a practical matter, feasiblity and ease of arterial access and catheterization are important qualities in the optimal animal model. In the rodent or rabbit—the most commonly used animal models for HCC—arterial access is either impossible or challenging, often requiring a surgical cut down.^7^, ^8^, ^14^ Once accessed, the vessels in these animals often are too small to accommodate catheters used in humans. While other animal models, such as the pig, do allow easier percutaneous vascular access for the testing of catheters and particles, the absence of liver cancer in these animals limits their utility.^15^, ^16^

To our knowledge, one prior study has demonstrated the feasibility of performing MRI and embolization in the woodchuck.^12^ However, this investigation was performed in nontumor models. Our work is performed in woodchuck tumor models; thus, in addition to demonstrating the feasibility of performing MRI and arterial access, we demonstrate tumor characteristic and pathologic assessment after embolotherapy.

We found that percutaneous arterial catheterization and liver embolization were able to be performed in the woodchuck HCC model using standard catheters and particles normally used in the treatment of human HCC. Pathological assessment after embolization using the protocol outlined in this manuscript allows for consistent microscopic tumor assessment for comparison of various IAT.

## CONFLICT OF INTEREST

None.

## AUTHOR CONTRIBUTIONS

AYK and SM designed the study. AYK, KK, and SM performed the procedures. JHY performed the imaging assessment. BUP and BK performed the pathologic assessment. AYK and DHF wrote the paper. All the authors read and approved the final manuscript.
